# Association between genome-wide copy number variation and arsenic-induced skin lesions: a prospective study

**DOI:** 10.1186/s12940-017-0283-8

**Published:** 2017-07-18

**Authors:** Muhammad G. Kibriya, Farzana Jasmine, Faruque Parvez, Maria Argos, Shantanu Roy, Rachelle Paul-Brutus, Tariqul Islam, Alauddin Ahmed, Muhammad Rakibuz-Zaman, Justin Shinkle, Vesna Slavkovich, Joseph H. Graziano, Habibul Ahsan

**Affiliations:** 1 Department of Public Health Sciences, University of Chicago, 900 E 57th Street, KCBD Bldg Room 6110, Chicago, IL 60637 USA; 2 Department of Environmental Health Sciences, Mailman School of Public Health, Columbia University, New York, NY USA; 3 Division of Epidemiology and Biostatistics, School of Public Health, University of Illinois, Chicago, IL USA; 4 University of Chicago Research Bangladesh, Dhaka, Bangladesh; 5 Department of Medicine, University of Chicago, Chicago, IL USA; 6 Department of Human Genetics, University of Chicago, Chicago, IL USA; 7 Cancer Research Center, University of Chicago, Chicago, IL USA

**Keywords:** Copy number variation, Arsenic, Skin lesion, lincRNA, Survival analysis, Gene-environment interaction

## Abstract

**Background:**

Exposure to arsenic in drinking water is a global health problem and arsenic-induced skin lesions are hallmark of chronic arsenic toxicity. We and others have reported germline genetic variations as risk factors for such skin lesions. The role of copy number variation (CNV) in the germline DNA in this regard is unknown.

**Methods:**

From a large prospectively followed-up cohort, exposed to arsenic, we randomly selected 2171 subjects without arsenic-induced skin lesions at enrollment and genotyped their whole blood DNA samples on Illumina Cyto12v2.1 SNP chips to generate DNA copy number. Participants were followed up every 2 years for a total of 8 years, especially for the development of skin lesions. In Cox regression models, each CNV segment was used as a predictor, accounting for other potential covariates, for incidence of skin lesions.

**Result:**

The presence of genomic deletion(s) in a number of genes (*OR5J2, GOLGA6L7P, APBA2, GALNTL5, VN1R31P, PHKG1P2, SGCZ, ZNF658*) and lincRNA genes (*RP11-76I14.1, CTC-535 M15.2, RP11-73B2.2*) were associated with higher risk [HR between 1.67 (CI 1.3-2.1) and 2.15 (CI 1.5-2.9) for different CNVs] for development of skin lesions independent of gender, age, and arsenic exposure. Some deletions had stronger effect in a specific gender (*ZNF658* in males, *SGCZ* in females) and some had stronger effect in higher arsenic exposure (lincRNA *CTD-3179P9.1*) suggesting a possible gene-environment interaction.

**Conclusion:**

This first genome-wide CNV study in a prospectively followed-up large cohort, exposed to arsenic, suggests that DNA deletion in several genes and lincRNA genes may predispose an individual to a higher risk of development of arsenic-induced skin lesions.

**Electronic supplementary material:**

The online version of this article (doi:10.1186/s12940-017-0283-8) contains supplementary material, which is available to authorized users.

## Background

Over 100 million individuals worldwide are exposed to arsenic through drinking water, including 28–57 million in Bangladesh [1] and 13 million in the United States [2]. Arsenic is a class-I human carcinogen, and chronic exposure to high levels (>300 μg/L) is associated with increased risk for a wide array of diseases including cancers of the lung [3], liver [4], bladder [5, 6], kidney [7, 8], as well as neurological [9], metabolic [10] and cardiovascular [11–15] diseases, skin lesions [16–19] and maternal health [20]. Chronic arsenic exposure through drinking water is associated with an increase in mortality [21]. Most arsenic-related cancers have a long latency period, but arsenic-induced skin lesions appear relatively early [6, 22]. Moreover, hyperkeratosis may be considered as precursors to arsenic-induced basal and squamous cell carcinoma of skin [23]. Smith and Steinmaus [24] have reviewed a large number of epidemiologic studies, mostly case–control, showing the association between arsenic exposure and skin lesions.

Previously, using case–control design in a Bangladeshi population, in the first genome wide association study (GWAS) in arsenic, our group found some single nucleotide polymorphisms (SNP) to be associated with arsenic metabolism [25]. Using clinical follow-up data of one of the largest cohorts exposed to arsenic through drinking water, our group also presented evidence that a higher risk of arsenic-induced skin lesions was found in the male gender, higher age and higher arsenic exposure [16]. We have clinically followed-up a large cohort in Bangladesh exposed to different levels of arsenic through drinking water [26, 27]. A large number of subjects from this cohort were randomly selected for GWAS and we have done SNP genotyping using oligonucleotide based arrays from whole blood DNA collected at baseline. We have previously demonstrated the utility of these oligonucleotide based arrays to detect and interpret copy number (CN) changes in clinical samples [28]. A copy number variant (CNV) is a term collectively used to describe gains or losses of DNA sequence >1 kb in length. These may have a direct effect on transcription and transcriptional regulation, which in turn may be a cause for disease susceptibility and phenotypic variation [29].

It may be noted that CNVs represent a large class of genomic variation that was not well studied in the past, but is now gaining the attention of many investigators [30–32]. CNVs have already been reported to be associated with autism [33], schizophrenia [34, 35] and Crohn’s disease [36, 37]. In a tumor tissue based study, DNA losses at chromosomes 1q21.1, 7p22.3, 9q12 and 19q13.31 have been reported in arsenic-related lung squamous cell carcinoma [38]. In this study, we evaluate whether there is any association of CNVs in germ line DNA in the development of arsenic-induced skin lesion. To our knowledge, we present the first paper addressing the role of germ line CNVs in the development of arsenic-induced skin lesions in a population exposed to arsenic through drinking water.

## Methods

### Study subjects

The Health Effects of Arsenic Longitudinal Study (HEALS) was designed to investigate the health effects of arsenic exposure through drinking water in a population-based sample of adults in Araihazar, Bangladesh [26]. The study methods have been described previously [26]. The study protocol was approved by the Institutional Review board of The University of Chicago, Columbia University, and the Bangladesh Medical Research Council. Informed consent was obtained from all participants. At the start of the study, we identified 12,050 eligible individuals for recruitment from the enumerated total of approximately 65,000 residents in the study area. Between October 2000 and May 2002, we recruited married individuals aged 18–75 years who had been residing in the study area for at least 5 years. A total of 11,746 men and women enrolled into the HEALS cohort. At the baseline interview, trained study physicians blinded to the arsenic concentrations in participant’s drinking water conducted in-person interviews and clinical evaluations including skin examination. They collected spot urine and blood samples from the participants according to a structured protocol. Participants were contacted for a follow-up examination at an interval of 2 years, which followed the same baseline protocol. For this study, we have utilized the biological samples collected at baseline, the clinical skin evaluation data at baseline and subsequent four biennial follow-ups (96 months). We randomly selected 2332 HEALS participants for genome-wide SNP genotyping. Among those 2332 HEALS participants, 2270 did not have any arsenic-induced skins lesion at the time of enrollment. In this study we only considered these 2270 HEALS participants with no prevalent skin lesions at baseline.

### Arsenic-induced skin lesions

A structured protocol was used to ascertain arsenic-induced skin lesions by trained study physicians. The study physicians recorded the presence or absence of melanosis (hyperpigmentation), leukomelanosis (hypopigmentation) or keratosis (thickening of skin typically on the palms and soles) [16]. All the study physicians were specially trained to diagnose arsenic-induced skin lesions. We ascertained incident skin lesion cases in a prospective fashion using a structured protocol [16]. For the present study, “skin lesion” was classified as presence of any of these three or a combination of them.

### Arsenic exposure

Well water arsenic (WAs) concentrations of all 5966 wells in the study area were measured by graphite furnace atomic absorption spectrometry, with a detection limit of 5 μg/L. Samples below the limit of detection were subsequently reanalyzed by inductively coupled plasma mass spectrometry, with a detection limit of 0.1 μg/L [39]. In our present study, the 25th percentile, 50th percentile and 75th percentile of well water arsenic were 12 μg/L, 56 μg/L and 142 μg/L respectively (see Additional file 1: Table S1). It may be noted that the 25th percentile was close to the WHO guideline for arsenic in drinking water (10 μg/L) and the 50th percentile was close to the Bangladesh national standard for arsenic in drinking water (50 μg/L). The urinary total arsenic concentration was measured by graphite furnace atomic absorption spectrometry [40]. Urinary creatinine was measured by a colorimetric method based on the Jaffe reaction described by Heinegard and Tiderstrom [41]. The urinary arsenic was measured from a spot urine collection. To take into account the hydration status, we used the urinary arsenic creatinine ratio (UACR) as measure of arsenic exposure. The log_2_-transformed UACR showed strong correlation to the log_2_-transformed well water arsenic concentration (*r* = 0.66, see Additional file 2: Figure S1).

### Illumina SNP array

DNA was extracted from whole blood using the Flexigene kit (Qiagen, USA). Quantification was done using a NanoDrop 1000. According to Illumina protocol 250 ng of DNA was genotyped on Cyto12 v2.1 chips with 294,602 markers (289,773 SNP markers and 4829 copy number markers) and read on the BeadArray Reader. Image data was processed in GenomeStudio software V2010.3. After cluster generation, the genotype calls, B allele frequency and log_2_R ratio (LRR) were calculated. In GenomeStudio, the copy number (CN) is expressed as log_2_R ratio (LRR). For a particular locus, if a DNA sample has 2 copies (CN = 2), the ratio of signal intensity in a test sample to reference (which also should have CN = 2) would be 1 and thus log_2_ of the ratio (LRR) would be log_2_ 1 = 0. In the same way, a sample with CN = 1 (intensity would be half compared to the reference) would have LRR = log_2_ 0.5 = −1, whereas a sample with CN = 4 (expected intensity would be double the reference) would have LRR = log_2_ 2 = 1.

### Quality control (QC) and filtering

We excluded the markers in sex chromosomes (*n* = 17,442). From the remaining 277,160 autosomal markers (272,663 SNP and 4497 copy number markers), a total of 3064 SNP markers (only 1.12%) were excluded due to poor performance. The remaining 274,096 autosomal markers were considered for further analysis. For each sample, standard deviation (SD) of LRR of these 274,096 autosomal markers was calculated. Another 70 samples with SD of LRR >0.28 and 29 samples with call rate < 99.0% were excluded (see Additional file 3: Figure S2). Thus, we finally used high quality genomic data from a total of 2171 HEALS participants who did not have any arsenic-induced skin lesion at baseline and were prospectively followed-up for development of any arsenic-induced skin lesions. Characteristics of the study subjects are shown in Additional file 1: Table S1.

### Genome-wide CN analysis

GenomeStudio generated LRR data was imported into Partek genomic suite and transformed to CN data in linear scale [42]. Standard Principal Component Analysis (PCA) and a sample histogram were generated as part of QC. After obtaining the CN value for each locus, to identify the genomic regions with amplification, normal CN or deletion, we used a genomic segmentation algorithm [42]. By “genomic region” in a particular sample we mean a stretch of DNA showing amplification or deletion. A genomic region with CN variation in one sample may or may not fully overlap with a genomic region in another sample. By amplification or deletion “segment”, we mean the stretch of amplified or deleted segment that is common in at least 5% of the samples in this paper.

For the data from the Cyto12 v2.1 chips, the genomic segmentation was done with a setting of a minimum of 6 markers, signal to noise 0.3, and *p*-value threshold of 0.001 for two neighboring regions having significantly differing means. A genomic region was considered as amplified if the geometric mean CN was >2.3 and a deletion if the mean was <1.7. We restricted the analysis for the autosomes only (2.8% of the segments had CN > 2.3, 22.12% had CN <1.7 and the 75.0% had a copy number between 1.7-2.3). The length of a genomic segment was calculated from the genomic location of the start and end SNP for that genomic segment. In this paper, we reported a genomic segment in a sample to have amplification (0:no amplification, 1:amplification) or deletion (0:no deletion, 1:deletion) only if it was at least 5 kb in size and the geometric mean of the CN within the genomic boundary of the segment for that particular sample was >2.3 or <1.7 respectively. We used the CN status for each segment as a binary predictor for development of arsenic-induced skin lesions (0: no skin lesion, 1: skin lesion) in survival analysis.

### Statistical analysis

To compare the continuous variables (e.g. age, UACR, WAs, BMI,), we used one-way analysis of variance (ANOVA). For the categorical variables we used chi-square tests. We used both Kaplan-Meier curves and Cox Regression analysis. In survival analysis, the event was defined as any skin lesion detected during the follow-up visits. The time (months) was calculated from the enrollment to the first detection of any skin lesion (for “event”) and from the enrollment to the last follow-up (for the “censored”). We dichotomized the continuous variables by median value – age (0: age = <median 38, vs. 1: age > median), UACR (0: urinary arsenic creatinine ratio = <median 192 μg/g of creatinine vs. 1: >median). In Cox regression, we used the following model:

$$ \ln \left(\frac{H(t)}{H_0(t)}\right)={b}_1 Gene+{b}_2 Gender+{b}_3 Age+{b}_4 UACR $$

For the interaction models, we used the following:

$$ \ln \left(\frac{H(t)}{H_0(t)}\right)={b}_1 Gene+{b}_2 Gender+{b}_3 Age+{b}_4 UACR+{b}_5\times Gene\times Gender $$

$$ \ln \left(\frac{H(t)}{H_0(t)}\right)={b}_1 Gene+{b}_2 Gender+{b}_3 Age+{b}_4 UACR+{b}_5\times Gene\times UACR $$

Here, H(t)/H_0_(t) is the Hazard Ratio (HR). So in the model, if we use genomic deletion as a predictor (Gene), then the quantity exp.(b1) can be interpreted as the instantaneous relative risk of an event, at any time, for an individual with the genomic deletion present compared to an individual without the genomic deletion, given both the individuals are the same on all other covariates. For multiple testing, we used Bonferroni correction. The significance threshold was set at 4.4 E-5, which is 0.05/1135, the number of deletions identified and tested in this study.

## Result

Among the 2171 HEALS subjects (m = 1032, f = 1139) without arsenical skin lesions at baseline, a total of 301 male (29%) and 115 female (10%) subjects developed skin lesions during the 8 years of follow-up. Kaplan-Meir plots by gender and arsenic exposure are shown in Additional file 4: Figure S3. The higher incidence of skin lesions among male subjects [HR 2.76 (CI 2.2 – 3.4)] compared to females of similar age and arsenic exposure, is consistent with our previous report on shorter follow-up on a larger number of subjects from the same cohort [27]. Higher age of the individual (reflecting duration of arsenic exposure) and higher Urinary Arsenic Creatinine Ratio (UACR) (reflecting the level of arsenic exposure) were also associated with higher risk [HR 2.97 (CI 2.3-3.7) and HR1.6 (CI 1.3-1.9) respectively] for the development of arsenic-induced skin lesions (see Additional file 4: Figure S3 and Additional file 5: Figure S4).

**Fig. 1:**
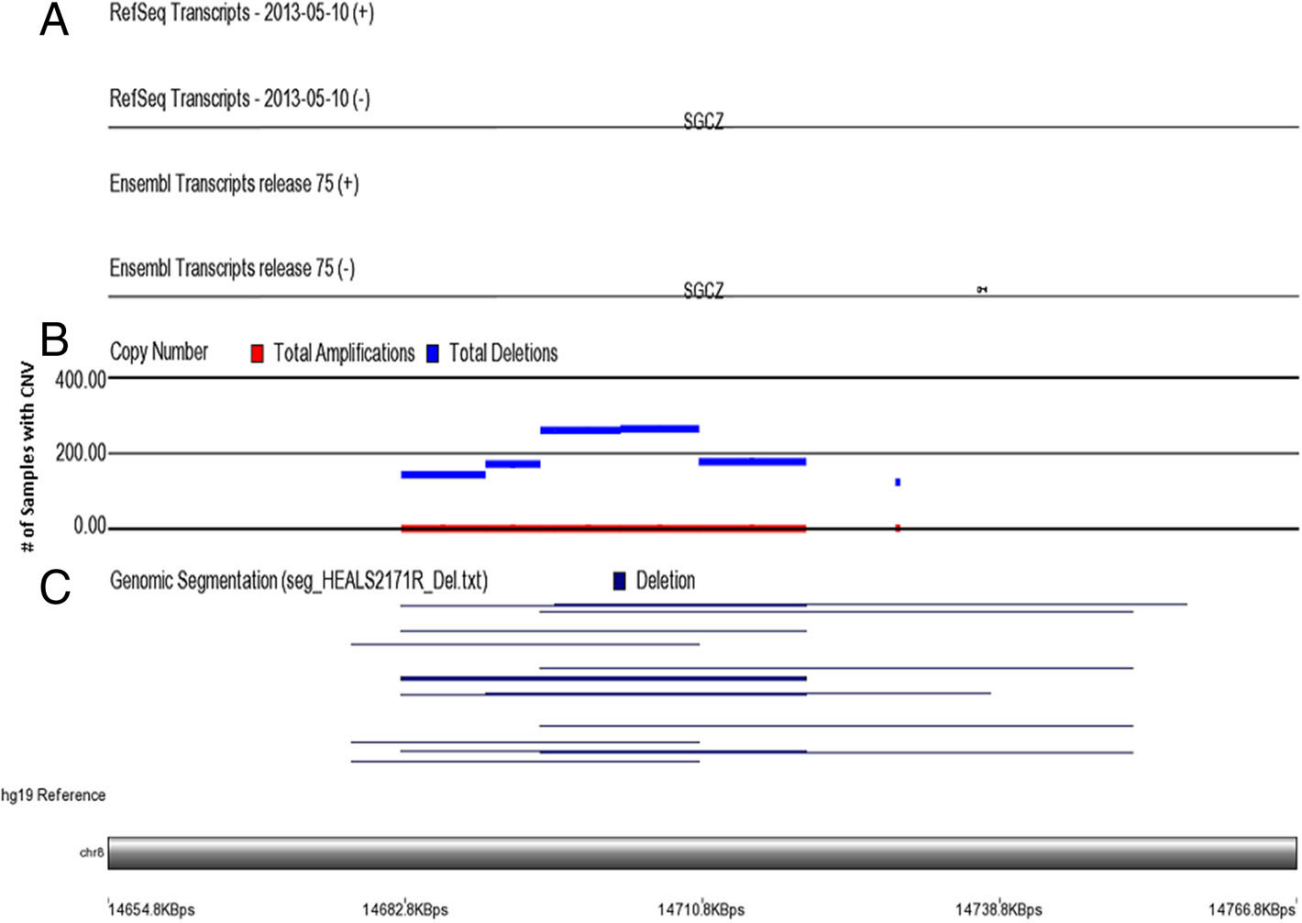
Copy number variants detected in 8p22 region. Top panel (panel **a**) shows mapping of the region to Refseq and Ensembl database; middle panel (panel **b**): y-axis shows the number of samples showing deletion; lower panel (panel **c**) shows different samples having deletion of different length (shown on x-axis), but the overlap is clearly seen

Structural variations were detected using Illumina Human Cyto12 v2.1 SNP chips. We identified a total of 1135 segments (at least 5 kb or longer in length), which showed CN loss <1.7 in at least 5% of the samples. We also found a total of 126 segments (at least 5 kb or longer in length), which showed CN gain >2.3 in at least 5% of the samples. Figure 1 shows an example of CNVs of a given region. The bottom-most panel (panel C) shows a deleted region in a number of samples. The genomic coordinates shown in x-axis correspond to the GRCh37/hg19 assembly. The length of deletion varies from sample to sample, however there are overlaps. In the middle panel (panel B), the y-axis shows the number of samples having deletion in that genomic region. So within that genomic region, there were five consecutive segments showing deletion. The top panel (panel A) shows annotation of the region as found in Refseq and Ensembl database.

To test the association between copy number loss/deletion and development of arsenic-induced skin lesions, we dichotomized the CN status of a segment as 0: no deletion and 1: deletion. Then, using Cox regression, we tested each of the 1135 segments for its potential role in the development of arsenic-induced skin lesions. We mentioned above that gender, age and UACR affect the risk of skin lesion. Therefore, along with the segments as a predictor, we also entered the gender (male vs. female), age (= < median 38 yrs. vs. >median) and UACR (= < median 192 μg/g of creatinine vs. >median) as covariates to see if the association(s) between segmental deletion and risk of skin lesion was independent of the covariates. Complete results from all these 1135 Cox regression models with HR and 95%CI of all the variables (genomic segment, gender, age and UACR) entered into the models are presented in Additional file 6: Table S2. We found a total of 24 segments covering 10 cytoband regions, deletion of which were significantly (Bonferroni *p* = <0.05) associated with higher risk of skin lesion development (HR ranging between 1.67 and 2.15 for different segments, see Table 1). The fact that multiple segments (mostly consecutive regions) within the same cytoband region (e.g. four successive segments in 5q34 cytoband region covering long intervening noncoding RNA (lincRNA) gene *CTC-535 M15.2*) were statistically significant for skin lesion risk, further strengthens our findings. Interestingly, out of these 10 cytoband regions, 3 of them (2q12.1, 5q34 and 7q11.21 shown in bold font in the Table 1) cover known lincRNA suggesting the possible significance of deletion of lincRNA as a risk factor for arsenic-induced skin lesions. For each CNV, the nearest gene is shown in Table 1. Our previous GWAS showed that two SNPs, rs9527 and rs11191659, were associated with arsenic metabolism [25]. However, conditioning on those two SNPs or smoking habit and chewing betel leaf did not change the effects of any of these genomic deletions on the development of skin lesions (see Additional file 7: Figure S5 and Additional file 8: Figure S6 respectively). Out of the 24 significant deletion segments shown in Table 1, 13 were reported in the database of genomic variants (DGV) (Table 1 shows the “distance to nearest reported CNV” = 0, for these regions), the 11 segments are not yet reported in the DGV (novel) (Table 1 shows the “distance to nearest reported CNV” > 0, for these regions), but these were within 82 kb distance from some other reported variants. Of the novel segments we found in the study that are associated with development of arsenic-induced skin lesions, 3 are from a known lincRNA *RP11-76I14.1* (2q12.1), and 4 are from another known lincRNA *CTC-535 M15.2* (5q34). The detail mappings are shown in Additional file 9: Figure S7.

**Table 1:** Cox regression analysis: Hazard Ratio (HR) for the development of arsenic-induced skin lesions by presence or absence of genomic deletions in different chromosomal locations. Combined analysis of total 2171 HEALS participants. Top genomic segments are sorted by chromosome

CNV genomic coordinates (GRCh37/hg19)	cytoband	length (bps)	Freq_Del	Nearest Gene	Distance to Nearest Gene (bps)^b^	HR (gene)^a^	95% CI (gene)		*p*-value	Bonferroni *p*-value	Nearest reported CNV in DGV database	Distance to Nearest reported CNV (bps)^c^
chr2:104,543,558-104,558,092	2q12.1	14,535	18.06%	**RP11-76I14.1 (+)**	0	1.70	1.36	2.13	4.35E-06	0.005	chr2:104,593,605..104760102	35,513
chr2:104,558,092-104,568,449	2q12.1	10,358	18.06%	**RP11-76I14.1 (+)**	0	1.70	1.36	2.13	4.11E-06	0.005	chr2:104,593,605..104760102	25,156
chr2:104,573,423-104,581,271	2q12.1	7849	18.70%	**RP11-76I14.1 (+)**	0	1.67	1.34	2.10	7.05E-06	0.008	chr2:104,593,605..104760102	12,334
chr3:102,717,330-102,770,447	3q12.3	53,118	6.54%	RNU1-43P (+)	108,357	2.11	1.53	2.91	5.08E-06	0.006	chr3:102,743,602..102752706	0
chr5:164,917,182-164,925,967	5q34	8786	5.62%	**CTC-535 M15.2 (+)**	0	2.15	1.55	2.98	4.62E-06	0.005	chr5:164,849,860..164874815	42,367
chr5:164,925,967-164,939,621	5q34	13,655	5.80%	**CTC-535 M15.2 (+)**	0	2.11	1.52	2.91	6.55E-06	0.007	chr5:164,849,860..164874815	51,152
chr5:164,939,621-164,956,122	5q34	16,502	5.85%	**CTC-535 M15.2 (+)**	0	2.10	1.52	2.90	7.39E-06	0.008	chr5:164,849,860..164874815	64,806
chr5:164,956,122-164,973,175	5q34	17,054	5.62%	**CTC-535 M15.2 (+)**	0	2.14	1.54	2.97	5.08E-06	0.006	chr5:164,849,860..164874815	81,307
chr7:151,708,046-151,713,498	7q36.1	5453	8.15%	GALNTL5 (+)	0	1.88	1.40	2.53	3.07E-05	0.035	chr7:151,794,196..151797877	80,698
chr7:151,713,498-151,719,077	7q36.1	5580	7.97%	GALNTL5 (+)	0	1.87	1.39	2.52	4.25E-05	0.048	chr7:151,794,196..151797877	75,119
chr7:62,764,248-62,830,796	7q11.21	66,549	15.98%	VN1R31P (−)	0	1.69	1.34	2.14	1.19E-05	0.014	chr7:62,830,337..62835635	0
chr7:62,835,371-62,889,537	7q11.21	54,167	15.80%	**RP11-73B2.2 (−)**	0	1.70	1.34	2.15	1.01E-05	0.012	chr7:62,864,828..62908102	0
chr7:62,889,537-62,990,599	7q11.21	101,063	16.12%	PHKG1P2 (+)	0	1.70	1.35	2.15	9.35E-06	0.011	chr7:62,985,473..63249879	0
chr8:14,696,759-14,702,931	8p22	6173	11.98%	SGCZ (−)	0	1.73	1.35	2.23	2.10E-05	0.024	chr8:14,697,130..14719288	0
chr8:14,702,931-14,710,309	8p22	7379	12.16%	SGCZ (−)	0	1.72	1.33	2.21	2.65E-05	0.030	chr8:14,697,130..14719288	0
chr8:14,720,385-14,737,759	8p22	17,375	5.62%	CTD-2023 J5.1 (−)	0	2.11	1.53	2.89	4.04E-06	0.005	chr8:14,680,345..14849094	0
chr9:40,824,400-40,839,148	9p13.1	14,749	11.05%	ZNF658 (−)	0	1.83	1.37	2.44	3.96E-05	0.045	chr9:40,832,700..41365793	0
chr9:40,857,839-40,876,494	9p13.1	18,656	13.54%	BX664608.1 (+)	44,448	1.72	1.33	2.23	3.88E-05	0.044	chr9:40,832,700..41365793	0
chr9:40,876,494-40,895,281	9p13.1	18,788	13.36%	BX664608.1 (+)	63,103	1.74	1.34	2.25	3.40E-05	0.039	chr9:40,832,700..41365793	0
chr11:55,900,420-55,952,826	11q12.1	52,407	11.79%	OR5J2 (+)	0	1.73	1.34	2.24	3.10E-05	0.035	chr11:55,962,241..55976332	9415
chr11:55,952,826-55,965,828	11q12.1	13,003	11.29%	OR8V1P (−)	0	1.75	1.34	2.26	2.84E-05	0.032	chr11:55,962,241..55976332	0
chr15:23,938,636-23,943,758	15q11.2	5123	7.46%	NDN (−)	6185	1.95	1.42	2.67	3.73E-05	0.042	chr15:23,973,556..23974955	29,798
chr15:29,061,004-29,089,826	15q13.1	28,823	21.10%	GOLGA6L7P (−)	0	1.71	1.32	2.21	3.87E-05	0.044	chr15:29,066,490..29069430	0
chr15:29,089,826-29,154,407	15q13.1	64,582	14.14%	APBA2 (+)	0	1.80	1.36	2.39	3.64E-05	0.041	chr15:29,093,623..29096097	0

^a^Hazard Ratio if genomic deletion is present; Cox regression model includes genomic segment, gender, age, UACR - all dichotomized variables. The linc RNA regions are shown in bold font. ^b^ genomic segments with “Distance to nearest gene” >0 indicates that the segment doesn’t cover the gene, but it is close to that gene. ^c^ genomic segments with “Distance to nearest reported CNV” = 0 indicates that the identified CNV overlaps with known CNV repoted in the DGV database

**Fig. 2:**
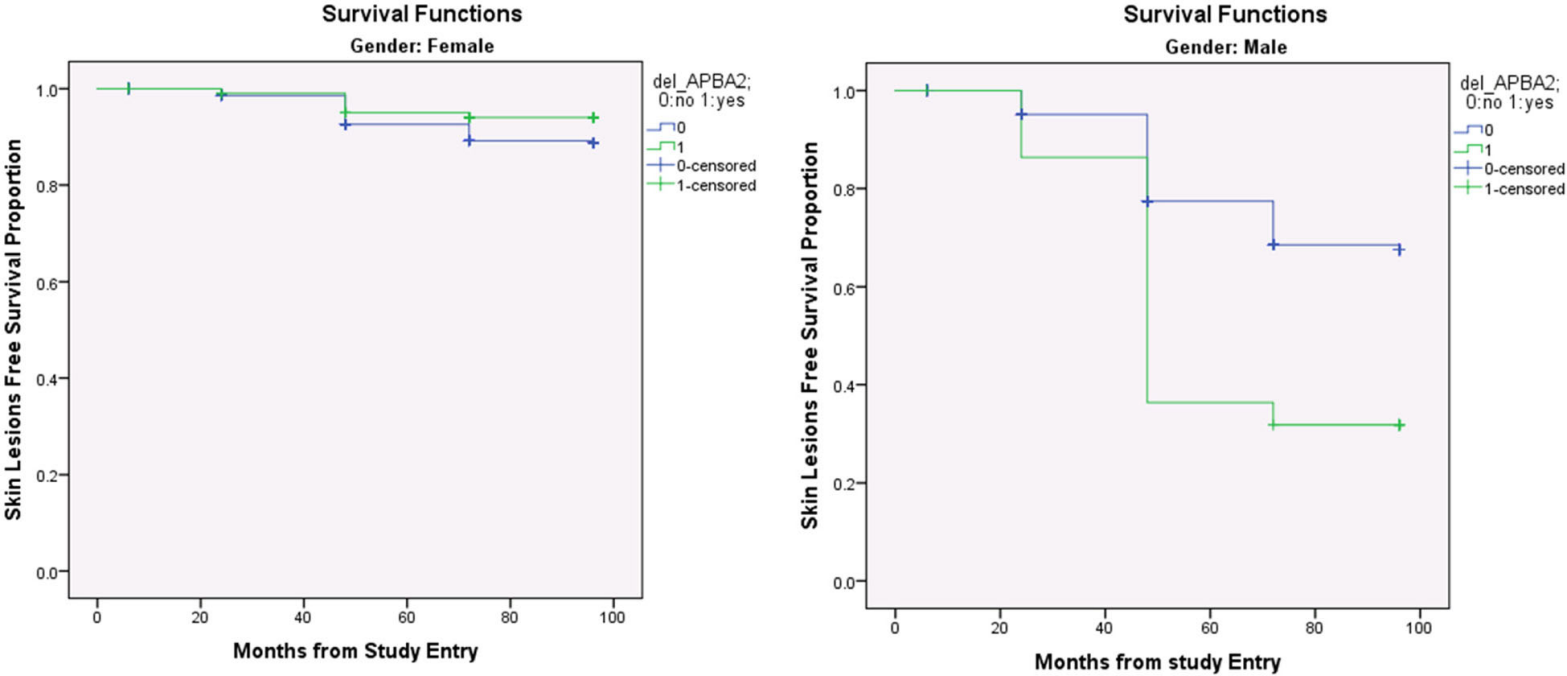
Kaplan-Meier curves: x-axis represents time in months; deletion in chromosome 15q13.1 covering the gene *APBA2* did not affect the development of arsenic-induced skin lesions in female subjects (*p* = 0.117, log rank test; shown on left), but significantly increased the risk in male subjects (*p* = 3.07E-05, log rank test; shown on right) exposed to arsenic through drinking water

In the next step, in Cox regression models, in addition to the previous covariates, we also entered an interaction term “segment x gender” to find out if the deletion of any of the segment(s) affected the risk of skin lesion differently in male and female subjects. That also allowed us to identify segments significant in male and female subjects separately. Table 2 shows the segments that achieved statistical significance after Bonferroni correction for multiple testing for male subjects. For example, the association of 9p12 deletion covering intronic region of the gene *ZNF658* and the development of arsenic-induced skin lesions is statistically significant in male subjects [HR 2.5, CI 1.7-3.7] and is stronger compared to that in female subjects [HR 1.3, CI 0.82-2.11] with interaction *p* = 0.03. This structural genomic variant has been reported in the DGV. Figure 2 shows the differential role of 15q13.3 deletion (*APBA2* gene) in males and females for the development of arsenic-induced skin lesions. The detail mappings of these regions are shown in Additional file 10: Figure S8.

**Table 2:** Cox regression analysis: Hazard Ratio (HR) for the development of arsenic induced skin lesions by presence or absence of genomic deletions in different chromosomal locations in male subjects

CNV genomic coordinates (GRCh37/hg19)	Cytoband	Length (bps)	Nearest Gene	Distance to Nearest Gene (bps)	HR (gene) Male subject^a^	95% CI (gene)		*p*-value (gene)	Bonferroni *p*-value (gene) Male subjects	interaction *p*-value	Nearest reported CNV in DGV database	Distance to Nearest reported CNV
chr9:40,768,295-40,779,600	9p13.1	11,306	ZNF658 (−)	0	2.42	1.62	3.62	1.76E-05	0.020	0.048	chr9:40,773,931..40940341	0
chr9:40,779,600-40,794,798	9p13.1	15,199	ZNF658 (−)	0	2.41	1.60	3.63	2.77E-05	0.031	0.054	chr9:40,781,674..40784428	0
chr9:40,794,798-40,812,799	9p13.1	18,002	ZNF658 (−)	0	2.55	1.71	3.79	4.01E-06	0.005	0.035	chr9:40,773,931..40940341	0
chr9:40,824,400-40,839,148	9p13.1	14,749	ZNF658 (−)	0	2.16	1.50	3.12	3.95E-05	0.045	0.180	chr9:40,832,700..41365793	0
chr15:29,214,310-29,222,222	15q13.1	7913	APBA2 (+)	0	2.59	1.64	4.09	4.53E-05	0.051	0.006	chr15:29,212,947..29288420	0
chr15:29,222,222-29,231,330	15q13.1	9109	APBA2 (+)	0	3.08	1.83	5.19	2.29E-05	0.026	0.001	chr15:29,212,947..29288420	0

^a^Hazard Ratio if genomic deletion is present; Cox regression model includes: genomic segment, age, gender, UACR and interaction term “genomic segment x gender”

Top genomic segments are sorted by chromosome

Table 3 presents the segments with significant effect in female subjects. The association of 8p22 deletion covering intronic region of the *SGCZ* gene and the development of arsenic-induced skin lesion is stronger in female subjects [HR 2.4, CI 1.6-3.7] compared to that in males [HR 1.4, CI 1.02-1.96] with interaction *p* = 0.04. This structural genomic variant has been reported in the DGV.

**Table 3:** Cox regression analysis: Hazard Ratio (HR) for the development of arsenic induced skin lesions by presence or absence of genomic deletions in different chromosomal locations in female subjects

CNV genomic coordinates (GRCh37/hg19)	Cytoband	Length (bps)	Gene	Distance to Nearest Gene (bps)	HR (gene) Female subject^a^	95% CI (gene)		*p*-value (gene)	Bonferroni *p*-value (gene) Female subjects	interaction *p*-value	Nearest reported CNV in DGV database	Distance to Nearest reported CNV
chr8:14,696,759-14,702,931	8p22	6173	SGCZ (−)	0	2.43	1.60	3.69	3.03E-05	0.034	0.057	chr8:14,697,130..14719288	0
chr8:14,702,931-14,710,309	8p22	7379	SGCZ (−)	0	2.46	1.63	3.72	1.91E-05	0.022	0.040	chr8:14,697,130..14719288	0

^a^Hazard Ratio if genomic deletion is present; Cox regression model includes: genomic segment, age, gender, UACR and interaction term “genomic segment x gender”

Top genomic segments are sorted by chromosome

**Fig. 3:**
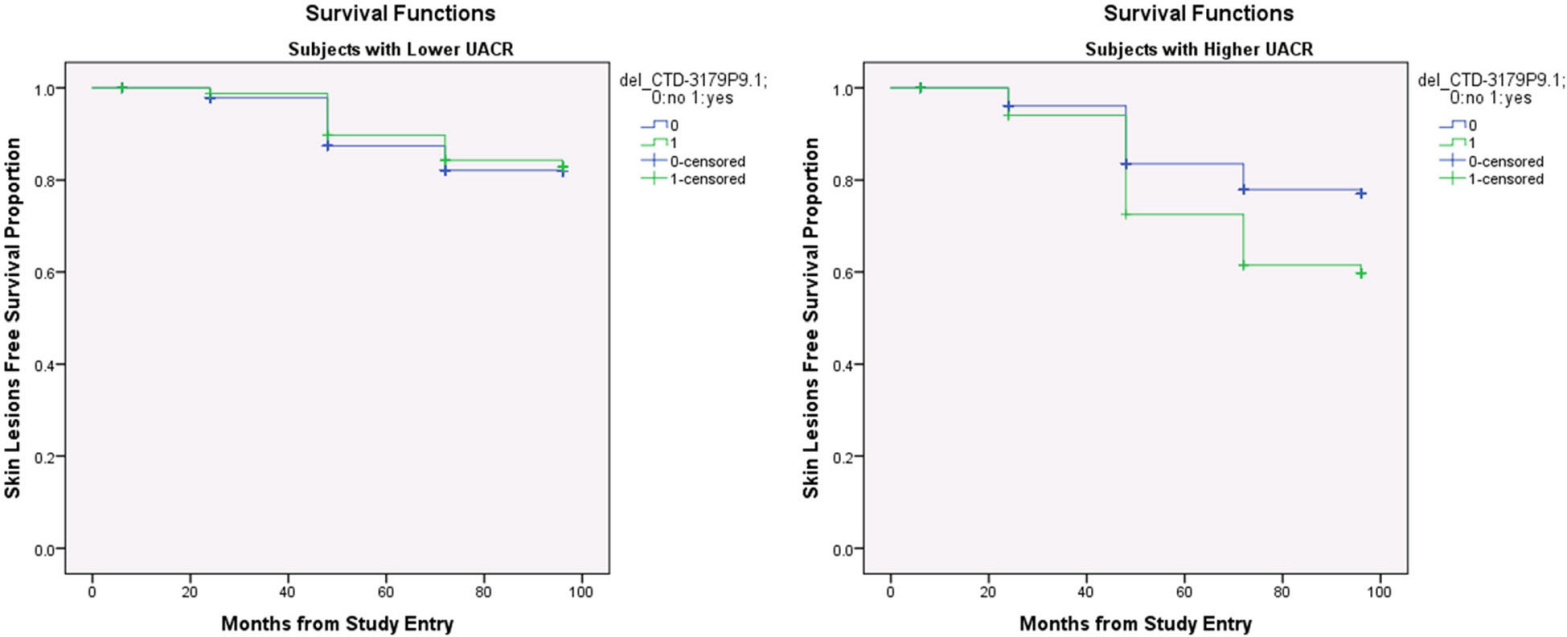
Kaplan-Meier curves: x-axis represents time in months; deletion in chromosome 5q23.1 covering the lincRNA *CTD-3179P9.1* did not affect the development of arsenic-induced skin lesions in subjects with lower UACR (< median 192 μg/g of creatinine, shown on left; *p* = 0.771, log rank test), but deletion of the same region significantly increased the risk in subjects with higher UACR (shown on right; *p* = 0.002, log rank test)

In the next step, in Cox regression analysis, in addition to gender, age, UACR, we also included an interaction term “segment x UACR” as predictors to find out if the deletion of any of the segment(s) affected the risk of skin lesion differently in subjects with high and low arsenic exposure (the Gene-Environment interaction). Results are presented in the Table 4. Most of these segments were in a lincRNA region. For example, among the group of individuals with high arsenic exposure (UACR > = median 192 μg/g of creatinine), those with deletion of 5q34 were at a 2.5 (CI 1.7-3.8) fold higher risk of skin lesion development compared to those without deletion. Among the group of individuals with low arsenic exposure, the corresponding HR was lower at 1.6 (CI 0.9-2.8). A similar effect was also seen for the deletion of chromosome 5q23.1 region (see Fig. 3). The detail mappings of these regions are shown in Additional file 11: Figure S9.

**Table 4:** Cox regression analysis: Hazard Ratio (HR) for the development of arsenic induced skin lesions by presence or absence of genomic deletions in different chromosomal locations in subjects with higher arsenic exposure (urinary arsenic creatinine ratio > = median value of 192 μg/g of creatinine)

CNV genomic coordinates (GRCh37/hg19)	cytoband	length (bps)	Nearest Gene	Distance to Nearest Gene (bps)^b^	HR (gene) high UACR^a^	95% CI (gene)		*p*-value (gene)	Bonferroni *p*-value (gene) high UACR	interaction *p*-value high UACR	Nearest reported CNV in DGV database	Distance to Nearest reported CNV (bps)^c^
chr2:104,543,558-104,558,092	2q12.1	14,535	**RP11-76I14.1 (+)**	0	2.05	1.52	2.76	2.41E-06	0.003	0.077	chr2:104,593,605..104760102	35,513
chr2:104,558,092-104,568,449	2q12.1	10,358	**RP11-76I14.1 (+)**	0	2.05	1.52	2.76	2.21E-06	0.003	0.075	chr2:104,593,605..104760102	25,156
chr2:104,573,423-104,581,271	2q12.1	7849	**RP11-76I14.1 (+)**	0	1.98	1.47	2.67	5.86E-06	0.007	0.103	chr2:104,593,605..104760102	12,334
chr3:102,717,330-102,770,447	3q12.3	53,118	RNU1-43P (+)	108,357	2.39	1.60	3.57	2.11E-05	0.024	0.348	chr3:102,743,602..102752706	0
chr5:117,349,895-117,357,592	5q23.1	7698	**CTD-3179P9.1 (+)**	0	2.36	1.57	3.56	3.80E-05	0.043	0.013	chr5:117,280,136..117451273	0
chr5:117,357,592-117,368,561	5q23.1	10,970	**CTD-3179P9.1 (+)**	0	2.35	1.56	3.53	4.37E-05	0.050	0.016	chr5:117,280,136..117451273	0
chr5:164,917,182-164,925,967	5q34	8786	**CTC-535 M15.2 (+)**	0	2.57	1.69	3.89	9.35E-06	0.011	0.224	chr5:164,849,860..164874815	42,367
chr5:164,925,967-164,939,621	5q34	13,655	**CTC-535 M15.2 (+)**	0	2.51	1.67	3.78	1.03E-05	0.012	0.214	chr5:164,849,860..164874815	51,152
chr5:164,939,621-164,956,122	5q34	16,502	**CTC-535 M15.2 (+)**	0	2.49	1.65	3.75	1.23E-05	0.014	0.223	chr5:164,849,860..164874815	64,806
chr5:164,956,122-164,973,175	5q34	17,054	**CTC-535 M15.2 (+)**	0	2.50	1.65	3.78	1.65E-05	0.019	0.287	chr5:164,849,860..164874815	81,307

^a^Hazard Ratio if genomic deletion is present; Cox regression model includes: genomic segment, age, gender, UACR and interaction term “genomic segment x UACR”. Rows representing linc RNA regions are shown in bold font

^b^genomic segments with “Distance to nearest gene” >0 indicates that the segment doesn’t cover the gene, but it is close to that gene. ^c^genomic segments with “Distance to nearest reported CNV” = 0 indicates that the identified CNV overlaps with known CNV repoted in the DGV

Top genomic segments are sorted by chromosome

We tested if a deletion of any of these segments is associated with arsenic exposure. In that line, in logistic regression analysis for each of those segments, we used the segment (0: no deletion, 1: deletion) as the dependant variable; and for the independent variables we entered the measure of arsenic exposure (= < median vs. >median) along with gender (0: female, 1: male). Our data suggests that neither higher UACR, nor higher well water (as measure of intensity of arsenic exposure) were associated with higher prevalence of deletion for any of the segments. For many of the segments, however, deletion was more frequently found among the female subjects compared to the males (see Additional file 12: Table S3).

We also looked for if a copy number gain/amplification was associated with development of arsenic-induced skin lesions. Accordingly, we dichotomized the CN status of each segment as 0: no amplification and 1: amplification. In the Cox regression models, we calculated the HR for each amplification segment by entering the segment (0 vs. 1) as the predictor for arsenic-induced skin lesions, along with the covariates, gender (male vs. female), age (= < median 38 years vs. >median) and UACR (= < median vs. >median). None of the segments with amplification showed significantly higher risk for development of arsenic-induced skin lesions after Bonferroni correction for multiple testing.

## Discussion

To our knowledge, this is the first large-scale genome-wide CN analysis to show from a prospectively followed-up cohort that the structural variation(s) in the germ line DNA may predispose an individual exposed to arsenic to develop arsenic-induced skin lesions. In tumor tissue, from arsenic-induced lung squamous cell carcinoma, deletion in chromosomal regions 1q21.1, 7p22.3, and 9q12 have been reported [38]. We were expecting to see some of the CNVs we found to overlap with those reported by Martinez et al. [38]. However, one of the explanations for the lack of overlap may be the fundamental difference between our study and the previous study. We have looked at CNV in blood DNA predisposing an individual to develop skin lesion. Our data does not suggest that the CNVs, we identified in the current study, were associated with arsenic exposure. On the other hand, the previous study focused at potentially “arsenic related” lung tissue specific and tumor-specific “somatic” CN change [38]. We have not yet examined the CN change in skin tissue from the arsenic-induced skin lesions biopsies. Hopefully in future we will be able to do that. But more importantly, as of now, we know very little about the functional significance of germline CNV; and have much to learn in chronic disease(s).

Previously, using case–control design, our group found some SNPs to be associated with arsenic metabolism [25]. Using clinical follow-up data in a larger cohort, our group also presented the evidence that higher risk of arsenic-induced skin lesion was found in male gender, increasing age and higher arsenic exposure [16, 27]. Now, we provide evidence that structural variation in the form of CN loss or deletion in certain genomic location(s) may have a role in the development of arsenic-induced skin lesions independent of gender, age, level of arsenic exposure and also independent of the SNPs related to arsenic metabolism. Our study is also the first to indirectly suggest the possible relationship between lincRNA and development of arsenic-induced skin lesions. The lincRNAs do not overlap exons of either protein-coding or other non-lincRNA types of genes. The role of lincRNAs are just being unveiled recently [43–48]. Thousands of lincRNAs are now known, however, many of their functions are still unknown [49].

Higher risk of development of arsenic-induced skin lesions among male individuals is reported by others as well [27, 50, 51]. As expected, higher UACR or higher well water arsenic content was related to higher risk of skin lesions. Reduction in arsenic exposure increases the odds that an individual with skin lesions would recover or show less severe lesions within 10 years [52]. Historically, in Bangladesh, the arsenic contamination in drinking water started after 1971 when the digging of deep wells started with an assumption that deep tube wells in rural areas would provide safe drinking water in terms of bacterial contamination [53]. Among the younger subjects (age = <median 38 yrs), the effect of higher UACR on development of skin lesions (HR2.6, CI 1.7-4.0) was stronger than its effect among the older subjects (HR1.39, CI 1.1-1.7). In most of the households, by culture, the male subjects (husbands) were older than the female subjects (wives), and male subjects usually consume higher volume of water than the female (3.8 L/day SD1.4 vs. 3.1 L/day SD 1.1, *p* = 3.3 × 10^−35^, in the present study). So even though they were consuming the water from the same source, perhaps the cumulative arsenic dosage was higher in males due to higher consumption and higher age (longer duration of exposure). But even after controlling for age, UACR, genetic markers (SNP and CNV), smoking and betel use, the male gender was strongly related to skin lesion development.

The structural variants, we are reporting in this paper to have significant association with the development of arsenic-induced skin lesions, have a frequency between 5% and 21% among the study population. However, for some of these variants that are previously reported in the DGV [31], the frequency was much different. For example, the deletion in 7q11.21 region: the deletion frequency ranges from 1 in 29,084 in a case–control study for developmental delay using arrays [54] to 35 in 2504 in a sequence-based study [32]; amplification/gain was reported as high as 121/270 [55]. These differences may be due to different population, different test platform, different disease entity of interest etc. The significant germ line CNVs we found in this study, to be related to arsenic-induced skin lesions, do not match with the CNVs in arsenic related lung cancer tissue [38].

To achieve genome-wide coverage, by design, the Illumina Cyto12 chip or most of the other commercially available SNP chips actually interrogate mostly SNPs in the inter-genic regions. Therefore, many of the GWAS hit SNPs are in fact far away from a gene. It is important to note the fact that, most of the statistically significant regions (20 out of 24) with deletion we found in this study, were overlapping or were within a gene. In other words, the list of significant deletion regions was enriched in genic regions. Only a few (4 out of 24) were located within inter-genic regions.

The utility of oligonucleotide based SNP chips for the detection of CN change and its interpretation in clinical samples was demonstrated by our group in the past [28]. In the past, we also validated the CN changes detected by similar SNP chips by comparing them with the results from a Luminex based multiplex assay for our other study (see Additional file 13: Figure S10). Our current study clearly suggests that there is some role of structural change in the genome (in the form of CN loss/deletion) in the development of arsenic-induced skin lesions, independent of the known clinical factors/parameters like age, sex, UACR level as well as the genotypes known to affect the arsenic metabolism.

One of the major strengths of this study is the long-term prospective follow-up and reasonably large sample size. The HRs of deletion(s) of these genomic segments for skin lesions may be slightly lower than the HRs of age, sex, but are not negligible. Over the last decade, we have been following up one of the largest cohorts exposed to arsenic [26] and depending on future availability of funding support, we have the opportunity to check the reproducibility of this novel finding in a larger and independent set of subjects.

We acknowledge the fact that the microarray platform used in this study (Cyto12) is not ultra-high density, the majority of the markers are intergenic and/or intronic in location and it does not have many markers in exonic regions. Functional characterization of the CNVs will be done in a future study. We did not have the source of RNA samples for these individuals to run gene expression to confirm the effects of deletion of lincRNA regions in the genomic DNA samples. For future studies, we will focus on this issue as well as tissue specificity.

## Conclusion

Our genome wide CN analysis study of a prospectively followed-up cohort of arsenic exposure from drinking water suggests that individuals with CNVs in several genomic locations are predisposed to higher risk of development of arsenic-induced skin lesions. A few of these CNVs include lincRNA gene regions. The findings need to be replicated in another independent study and functional characterization would be needed to better understand the underlying genetic mechanism.

## Additional files

Additional file 1: Table S1. Characteristics of HEALS participants selected for this study. None of them had arsenic induced skin lesion at baseline. (XLS 21 kb)

Additional file 2: Figure S1. Correlation of log_2_ transformed urinary arsenic creatinine ratio (UACR) and log_2_ transformed well water arsenic (WAs) concentration (*r* = 0.66, *p* = 3.7 E-280). (PPT 108 kb)

Additional file 3: Figure S2. QC of samples by standard deviation of Log R Ratio (LRR) and by SNP call rate of the array. (PPT 163 kb)

Additional file 4: Figure S3. Kaplan-Meir plots show (a) male subjects were at higher risk of developing arsenic-induced skin lesion than the female subjects (*p* = 4.5 E-35, log rank test; shown on left side) exposed to arsenic through drinking water; and (b) subjects with higher age (>median 38 years) were also at higher risk for development of skin lesion (*p* = 3.8 E-40; shown on right side) than those who were younger. X-axis represents time to event (months of follow-up after enrollment). (PPT 76 kb)

Additional file 5: Figure S4. Kaplan-Meir plots show (a) subjects with higher UACR (>median 192 μg/g of creatinine) were at higher risk of developing arsenic-induced skin lesion than those with lower UACR (*p* = 0.001, log rank test; shown on left side); (b) categorization by well water arsenic (WAs) also showed similar effect – higher risk in subjects drinking water with higher arsenic concentration (>median 56 μg/L) compared to those drinking water with lower arsenic concentration (*p* = 1.97 E-09, log rank test; shown on right side). X-axis represents time to event (months of follow-up after enrollment). (PPT 77 kb)

Additional file 6: Table S2. Results from all the 1135 Cox regression models with HR and 95%CI of all the variables (genomic segment, gender, age and UACR). (XLS 1116 kb)

Additional file 7: Figure S5. Conditioning on Arsenic metabolism SNPs did not show any effect on the HR of the genomic segments (adjusted for gender, age & UACR). (PPT 138 kb)

Additional file 8: Figure S6. Adjusting for smoking habit or use of betel leaf did not show any effect on the HR of the genomic segments (adjusted for gender, age & UACR). (PPT 141 kb)

Additional file 9: Figure S7. Detail mapping of the CNV regions (presented in Table 1), predisposing to significantly higher risk for development of arsenic-induced skin lesions. (PDF 201 kb)

Additional file 10: Figure S8. Detail mapping of the CNV regions showing interaction with gender (presented in Tables 2 and 3) for higher risk of developing arsenic-induced skin lesions. (PDF 234 kb)

Additional file 11: Figure S9. Detail mapping of the CNV regions showing interaction with higher arsenic exposure (presented in Table 4) for higher risk of developing arsenic-induced skin lesions. (PDF 239 kb)

Additional file 12: Table S3. Frequency of the significant deleted segments in female and male subjects. (XLS 24 kb)

Additional file 13: Figure S10. Correlation between copy number of a genomic region derived from oligonucleotide SNP chip and fluorescent intensity derived from Luminex based assay. (PPT 159 kb)

## Abbreviations

ANOVA: Analysis of variance
CI: Confidence interval
CN: Copy number
CNV: Copy number variation
GWAS: Genome wide association study
HEALS: Health Effects of Arsenic Longitudinal Study
HR: Hazard Ratio
lincRNA: Long intervening noncoding RNA
LRR: Log R ratio
QC: Quality control
SD: Standard deviation
SNP: Single nucleotide polymorphism
UARC: Urinary arsenic creatinine ratio
WAs: Water arsenic
