# Supplementary material for: Association between genome-wide copy number variation and arsenic-induced skin lesions: a prospective study
Source: Environ Health. 2017 Jul 18;16:75. doi: 10.1186/s12940-017-0283-8 (PMC5516382; doi:10.1186/s12940-017-0283-8)
Supplement: Supplementary file 2 — Correlation of log2 transformed urinary arsenic creatinine ratio (UACR) and log2 transformed well water arsenic (WAs) concentration (r = 0.66, p = 3.7 E-280). (PPT 108 kb) [file 12940_2017_283_MOESM2_ESM.ppt]

## Slide 1
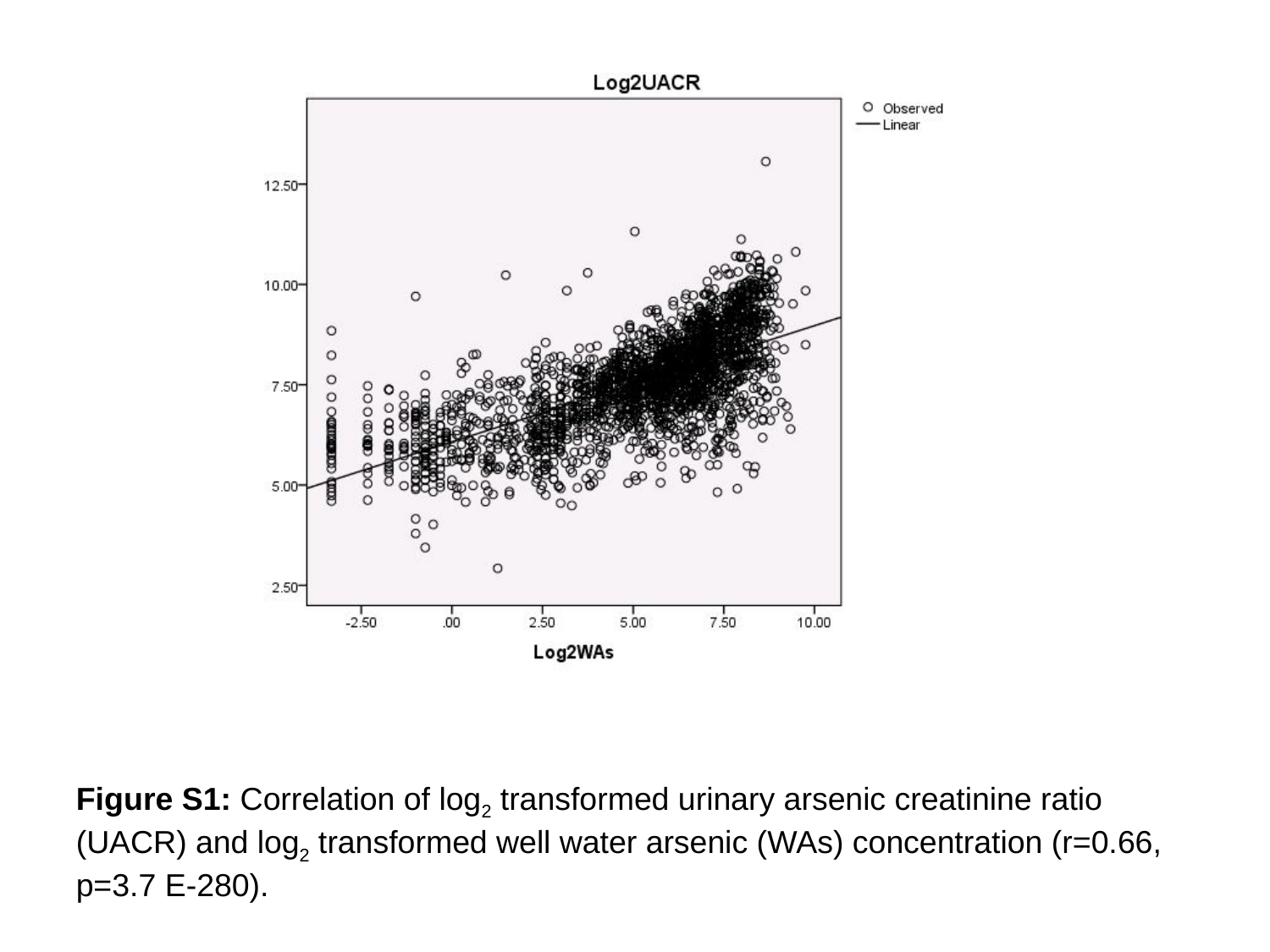

# Figure S1: Correlation of log2 transformed urinary arsenic creatinine ratio (UACR) and log2 transformed well water arsenic (WAs) concentration (r=0.66, p=3.7 E-280).
