# Supplementary material for: Association between genome-wide copy number variation and arsenic-induced skin lesions: a prospective study
Source: Environ Health. 2017 Jul 18;16:75. doi: 10.1186/s12940-017-0283-8 (PMC5516382; doi:10.1186/s12940-017-0283-8)
Supplement: Supplementary file 4 — Kaplan-Meir plots show (a) male subjects were at higher risk of developing arsenic-induced skin lesion than the female subjects (p = 4.5 E-35, log rank test; shown on left side) exposed to arsenic through drinking water; and (b) subjects with higher age (>median 38 years) were also at higher risk for development of skin lesion (p = 3.8 E-40; shown on right side) than those who were younger. X-axis represents time to event (months of follow-up after enrollment). (PPT 76 kb) [file 12940_2017_283_MOESM4_ESM.ppt]

## Slide 1
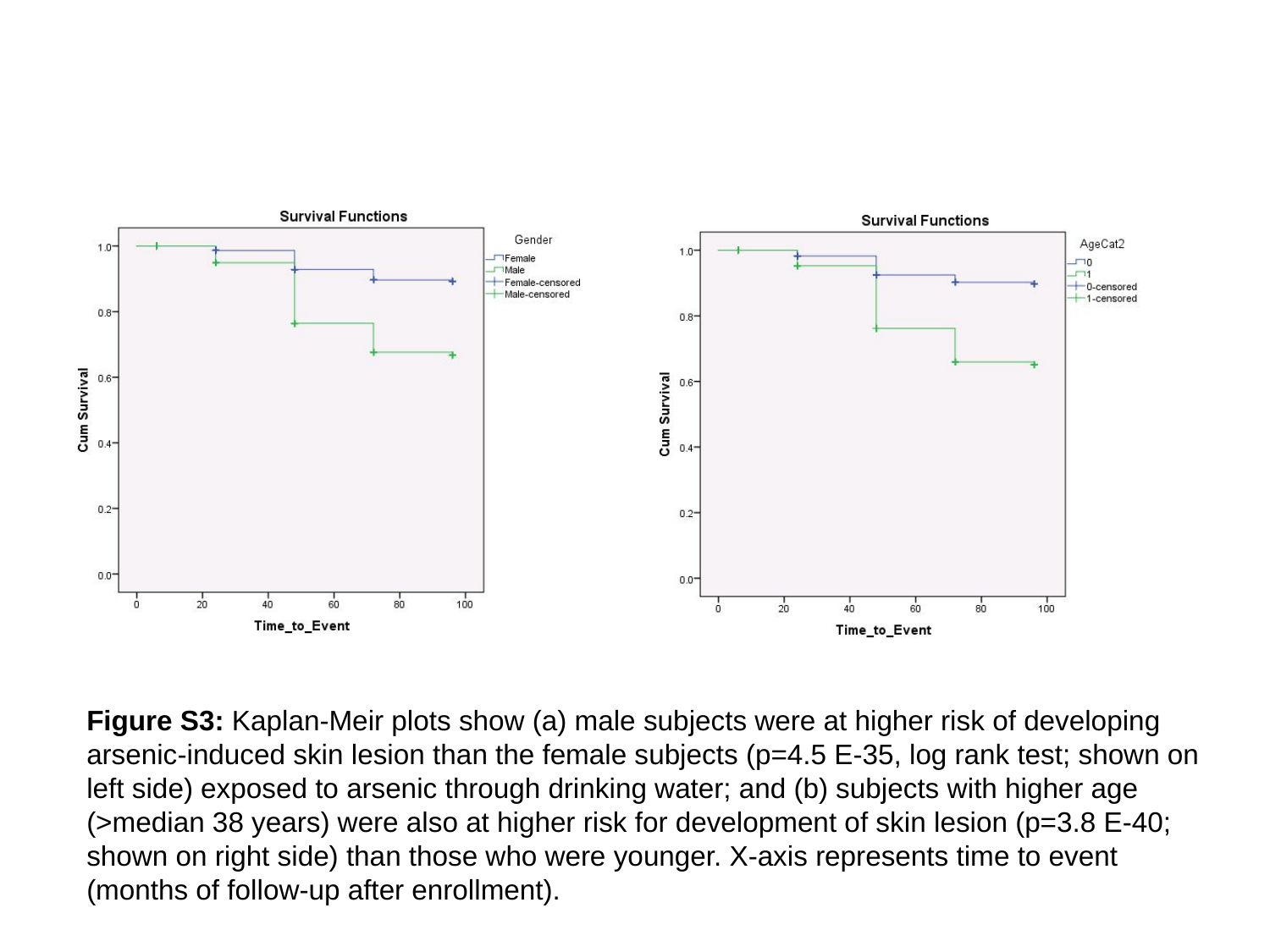

# Figure S3: Kaplan-Meir plots show (a) male subjects were at higher risk of developing arsenic-induced skin lesion than the female subjects (p=4.5 E-35, log rank test; shown on left side) exposed to arsenic through drinking water; and (b) subjects with higher age (>median 38 years) were also at higher risk for development of skin lesion (p=3.8 E-40; shown on right side) than those who were younger. X-axis represents time to event (months of follow-up after enrollment).
