# Supplementary material for: Association between genome-wide copy number variation and arsenic-induced skin lesions: a prospective study
Source: Environ Health. 2017 Jul 18;16:75. doi: 10.1186/s12940-017-0283-8 (PMC5516382; doi:10.1186/s12940-017-0283-8)
Supplement: Supplementary file 5 — Kaplan-Meir plots show (a) subjects with higher UACR (>median 192 μg/g of creatinine) were at higher risk of developing arsenic-induced skin lesion than those with lower UACR (p = 0.001, log rank test; shown on left side); (b) categorization by well water arsenic (WAs) also showed similar effect – higher risk in subjects drinking water with higher arsenic concentration (>median 56 μg/L) compared to those drinking water with lower arsenic concentration (p = 1.97 E-09, log rank test; shown on right side). X-axis represents time to event (months of follow-up after enrollment). (PPT 77 kb) [file 12940_2017_283_MOESM5_ESM.ppt]

## Slide 1
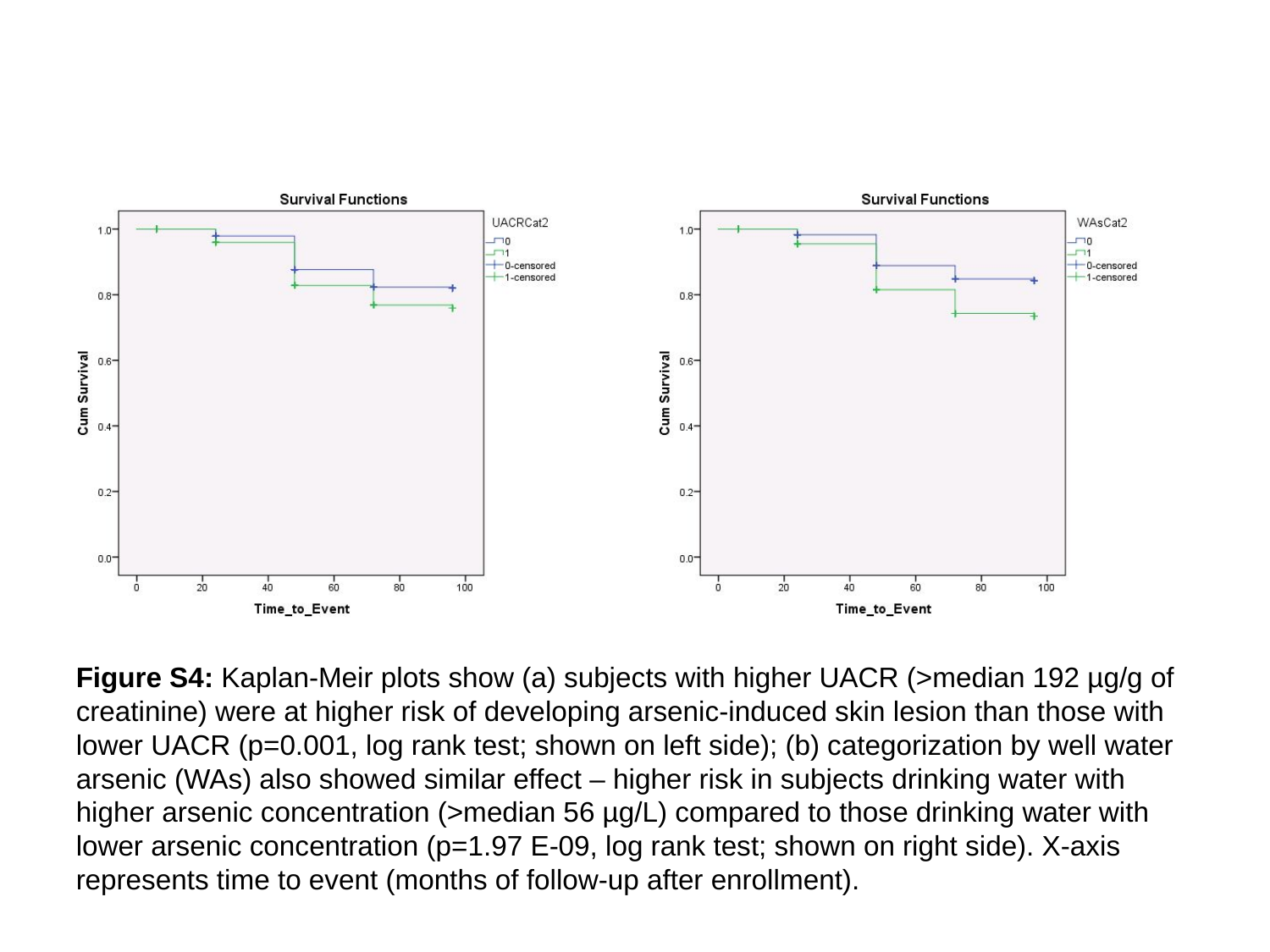

# Figure S4: Kaplan-Meir plots show (a) subjects with higher UACR (>median 192 µg/g of creatinine) were at higher risk of developing arsenic-induced skin lesion than those with lower UACR (p=0.001, log rank test; shown on left side); (b) categorization by well water arsenic (WAs) also showed similar effect – higher risk in subjects drinking water with higher arsenic concentration (>median 56 µg/L) compared to those drinking water with lower arsenic concentration (p=1.97 E-09, log rank test; shown on right side). X-axis represents time to event (months of follow-up after enrollment).
