# Supplementary material for: Association between genome-wide copy number variation and arsenic-induced skin lesions: a prospective study
Source: Environ Health. 2017 Jul 18;16:75. doi: 10.1186/s12940-017-0283-8 (PMC5516382; doi:10.1186/s12940-017-0283-8)
Supplement: Supplementary file 7 — Conditioning on Arsenic metabolism SNPs did not show any effect on the HR of the genomic segments (adjusted for gender, age & UACR). (PPT 138 kb) [file 12940_2017_283_MOESM7_ESM.ppt]

## Slide 1
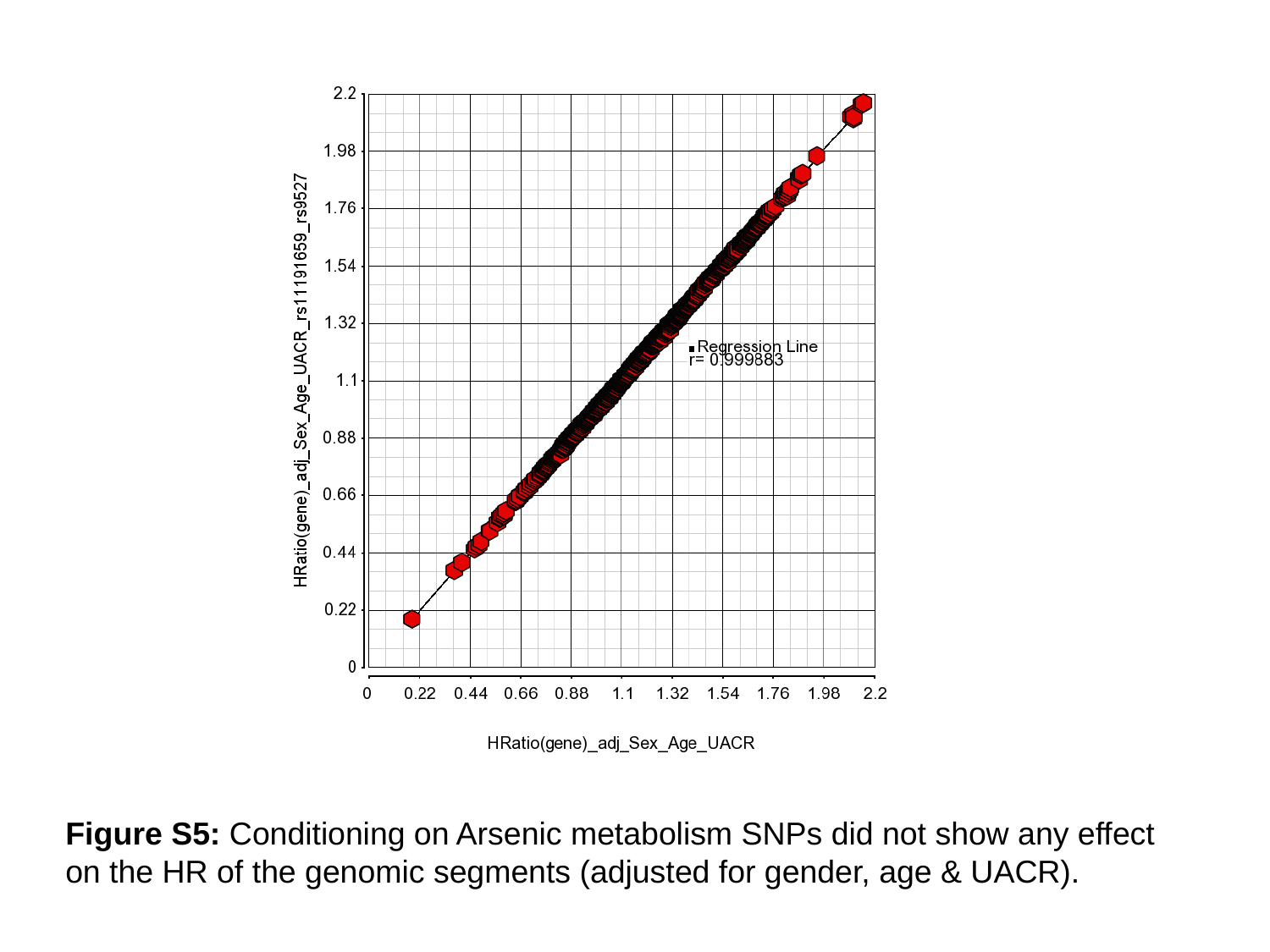

# Figure S5: Conditioning on Arsenic metabolism SNPs did not show any effect on the HR of the genomic segments (adjusted for gender, age & UACR).
