# Supplementary material for: Association between genome-wide copy number variation and arsenic-induced skin lesions: a prospective study
Source: Environ Health. 2017 Jul 18;16:75. doi: 10.1186/s12940-017-0283-8 (PMC5516382; doi:10.1186/s12940-017-0283-8)
Supplement: Supplementary file 8 — Adjusting for smoking habit or use of betel leaf did not show any effect on the HR of the genomic segments (adjusted for gender, age & UACR). (PPT 141 kb) [file 12940_2017_283_MOESM8_ESM.ppt]

## Slide 1
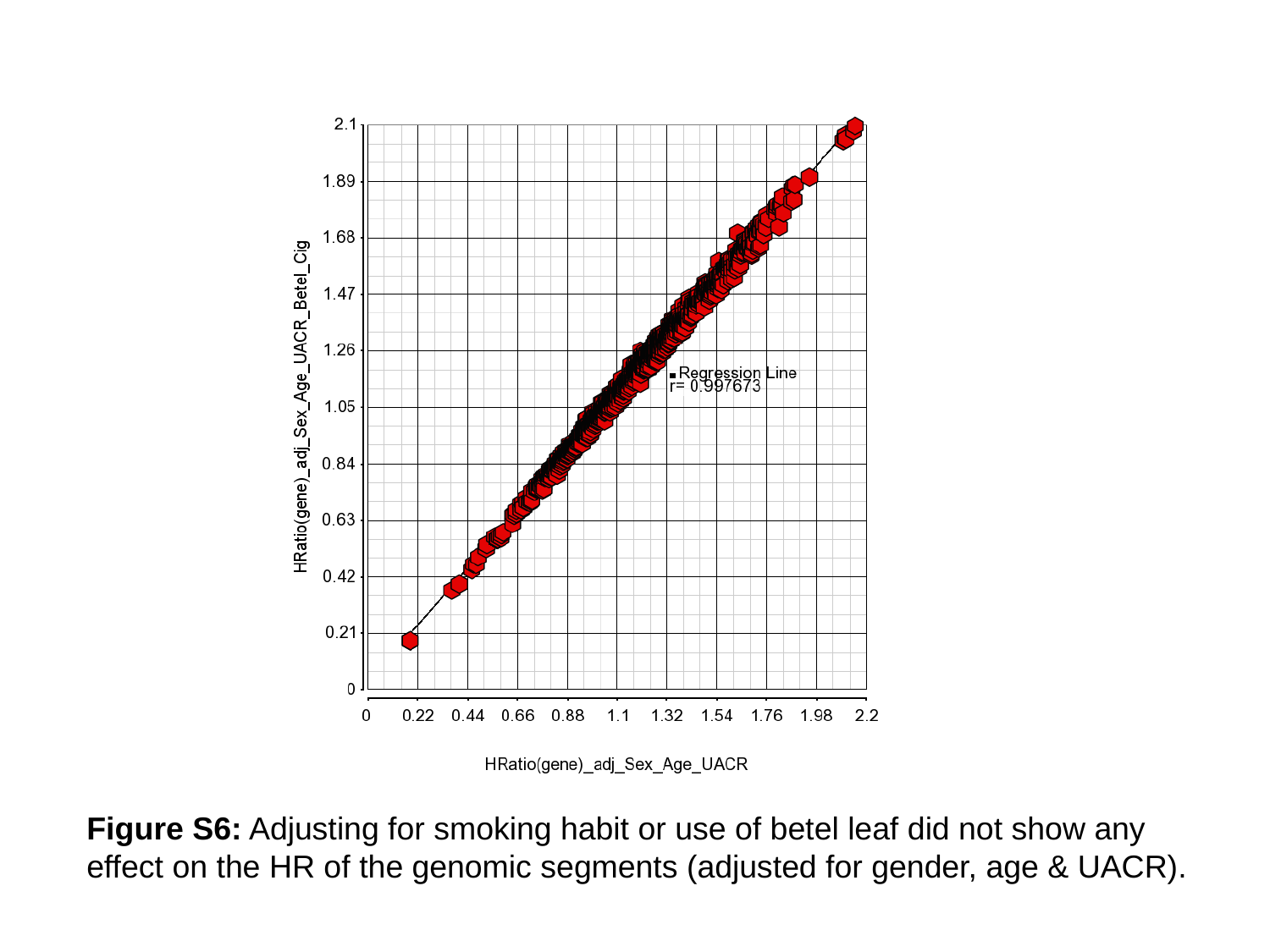

# Figure S6: Adjusting for smoking habit or use of betel leaf did not show any effect on the HR of the genomic segments (adjusted for gender, age & UACR).
