# Supplementary figures and images for: Association between genome-wide copy number variation and arsenic-induced skin lesions: a prospective study
Source: Environ Health. 2017 Jul 18;16:75. doi: 10.1186/s12940-017-0283-8 (PMC5516382; doi:10.1186/s12940-017-0283-8)

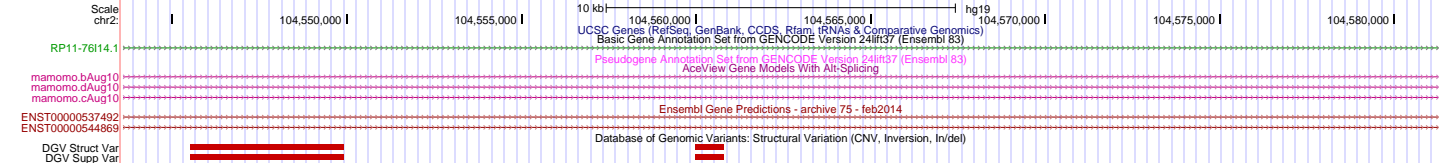

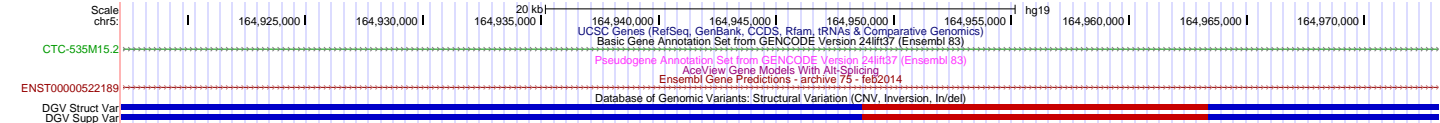

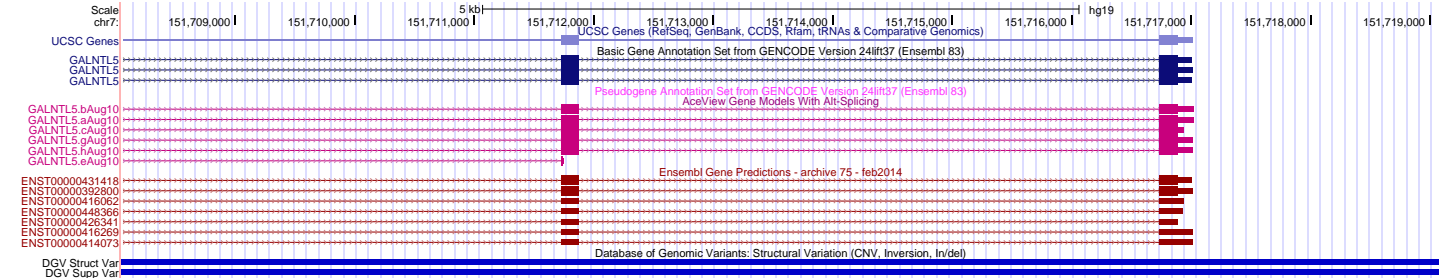

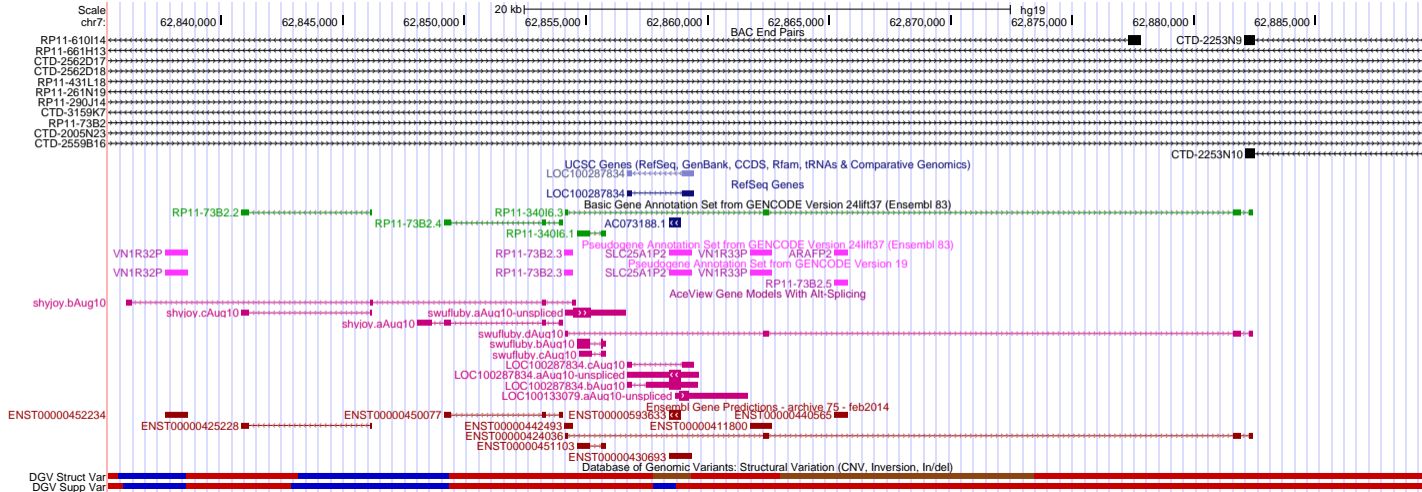

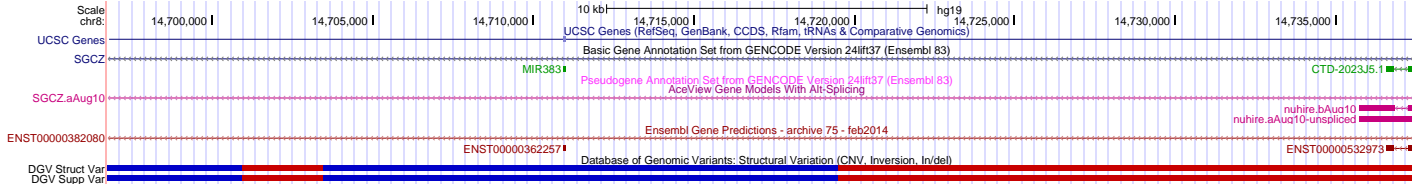

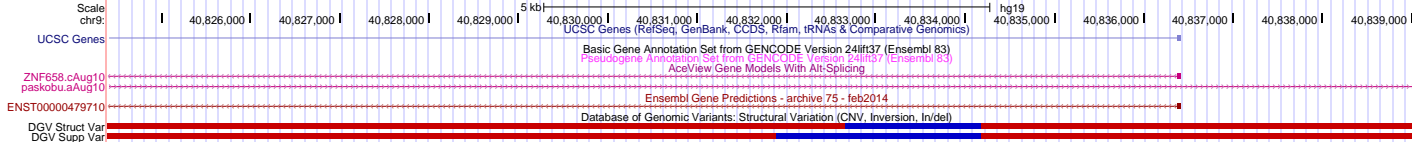

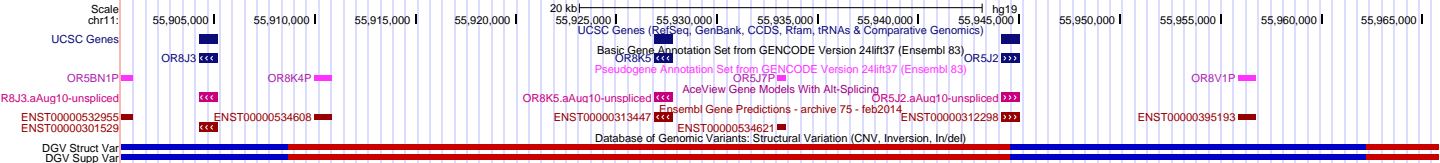

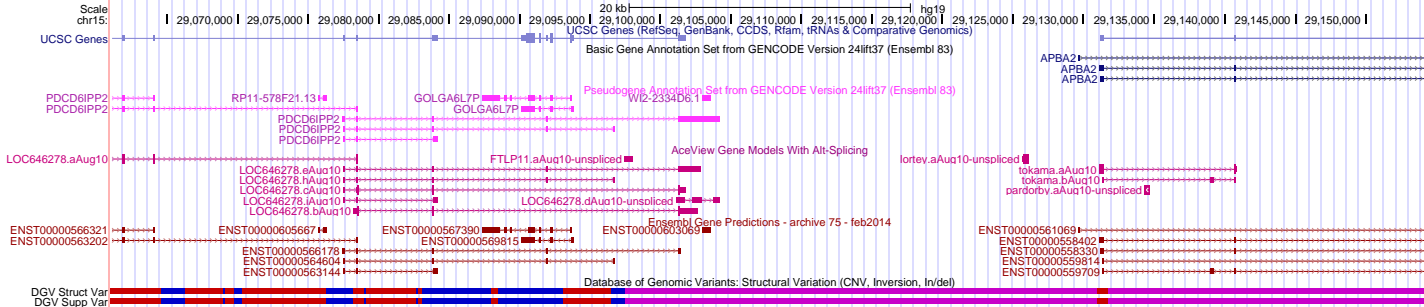

Supplement: Supplementary file 9 — Detail mapping of the CNV regions (presented in Table 1), predisposing to significantly higher risk for development of arsenic-induced skin lesions. (PDF 201 kb) [file 12940_2017_283_MOESM9_ESM.pdf]

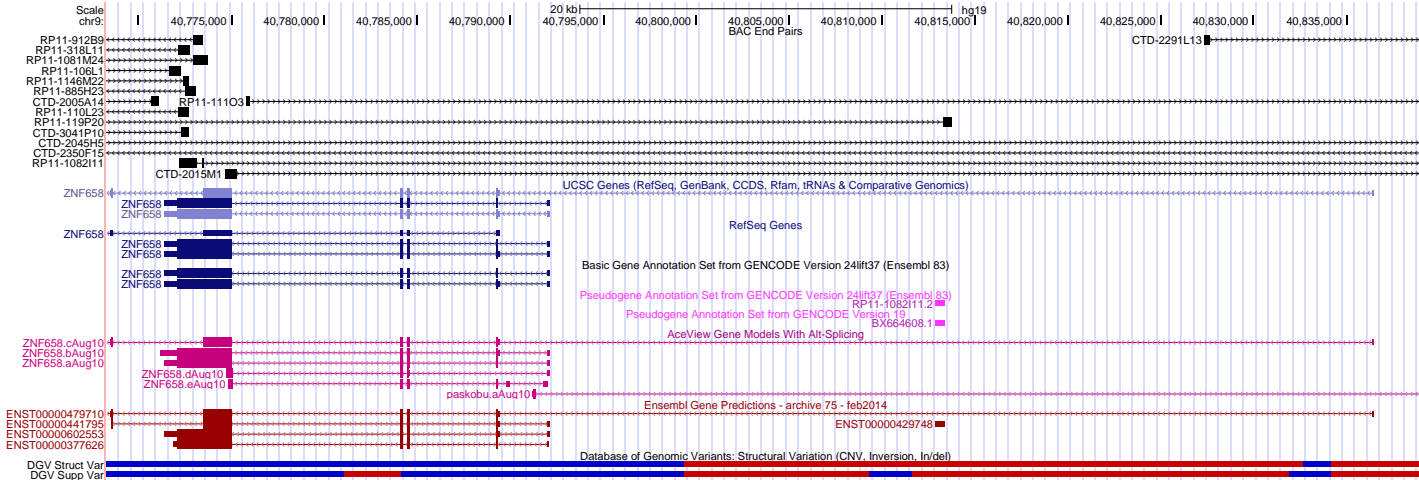

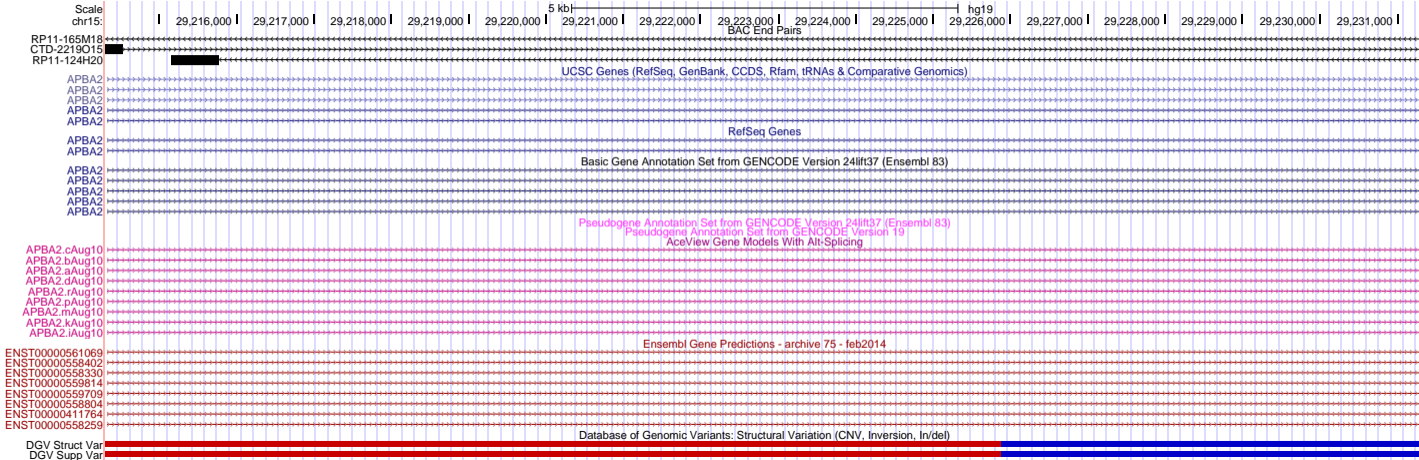

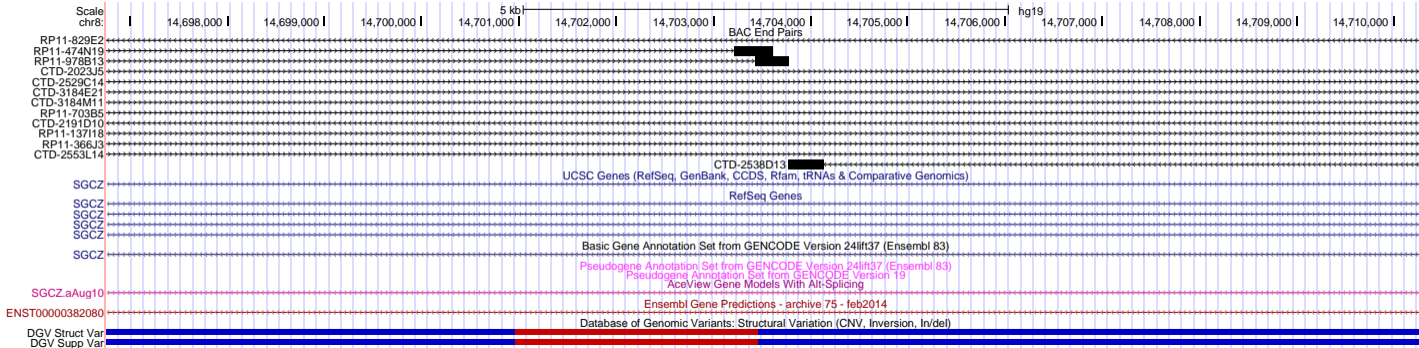

Supplement: Supplementary file 10 — Detail mapping of the CNV regions showing interaction with gender (presented in Tables 2 and 3) for higher risk of developing arsenic-induced skin lesions. (PDF 234 kb) [file 12940_2017_283_MOESM10_ESM.pdf]

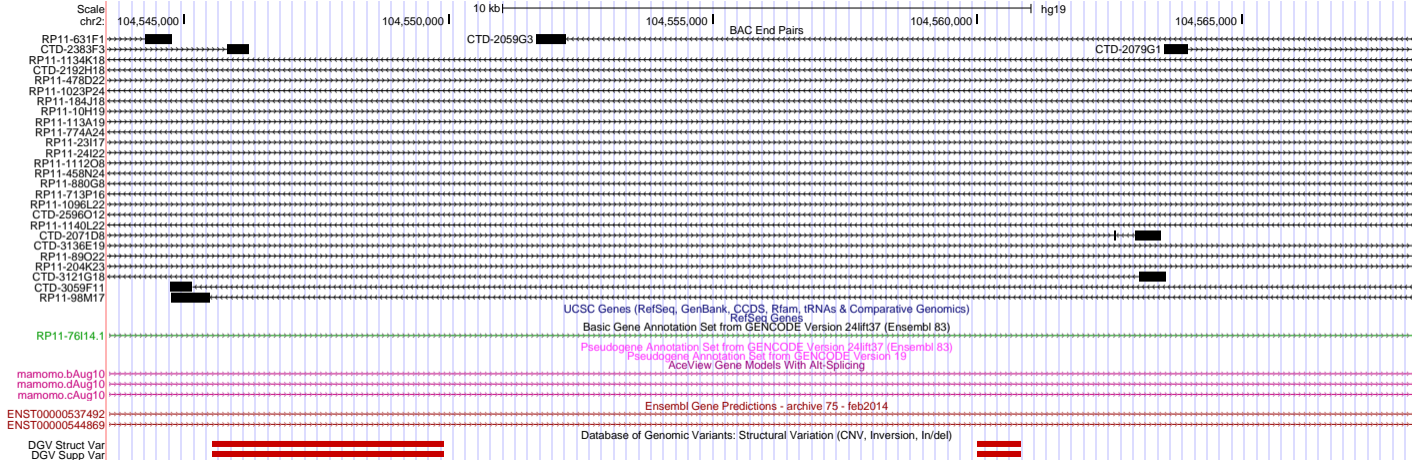

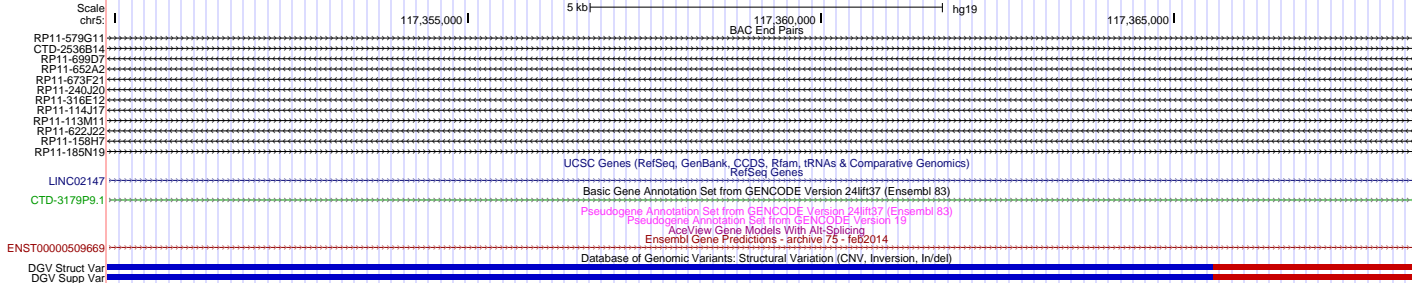

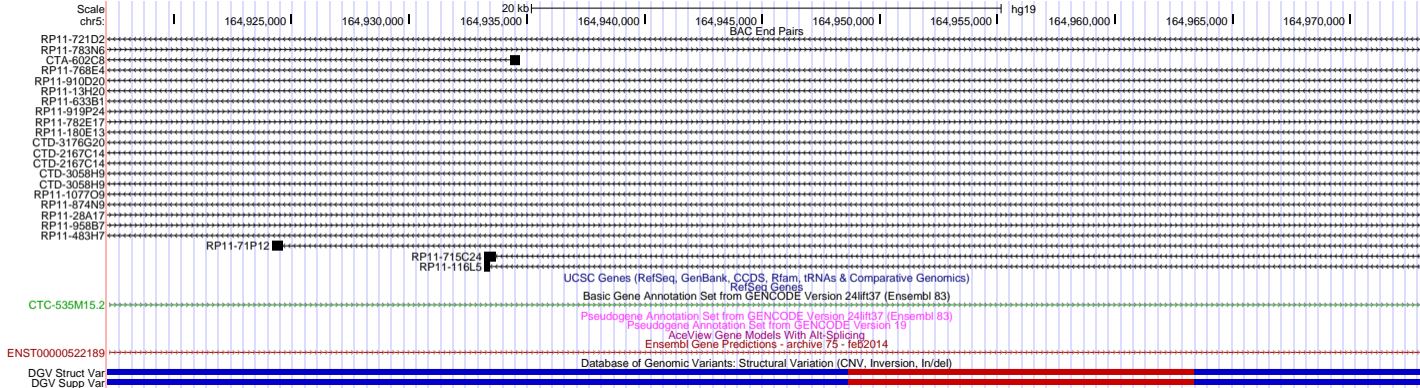

Supplement: Supplementary file 11 — Detail mapping of the CNV regions showing interaction with higher arsenic exposure (presented in Table 4) for higher risk of developing arsenic-induced skin lesions. (PDF 239 kb) [file 12940_2017_283_MOESM11_ESM.pdf]
