# Supplementary material for: Association between genome-wide copy number variation and arsenic-induced skin lesions: a prospective study
Source: Environ Health. 2017 Jul 18;16:75. doi: 10.1186/s12940-017-0283-8 (PMC5516382; doi:10.1186/s12940-017-0283-8)
Supplement: Supplementary file 13 — Correlation between copy number of a genomic region derived from oligonucleotide SNP chip and fluorescent intensity derived from Luminex based assay. (PPT 159 kb) [file 12940_2017_283_MOESM13_ESM.ppt]

## Slide 1
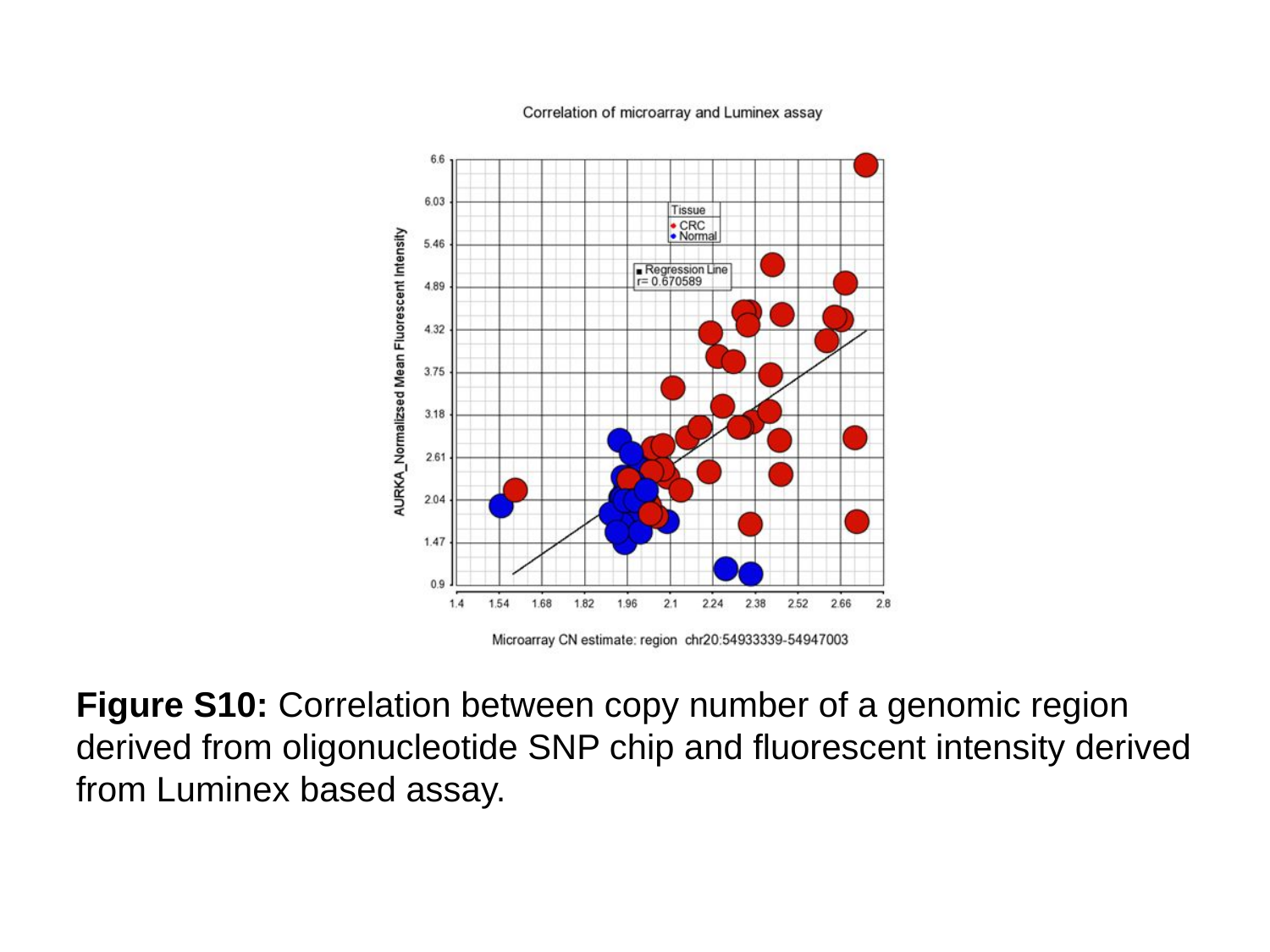

# Figure S10: Correlation between copy number of a genomic region derived from oligonucleotide SNP chip and fluorescent intensity derived from Luminex based assay.
